# Characterisation and Colour Response of Smart Sago Starch-Based Packaging Films Incorporated with *Brassica oleracea* Anthocyanin

**DOI:** 10.3390/membranes12100913

**Published:** 2022-09-21

**Authors:** Nurul Husna Che Hamzah, Nozieana Khairuddin, Ida Idayu Muhamad, Mohd Ali Hassan, Zainab Ngaini, Shahrul Razid Sarbini

**Affiliations:** 1 Department of Science and Technology, Faculty of Humanities, Management, and Science, Universiti Putra Malaysia, Bintulu Sarawak Campus, Bintulu 97008, Malaysia; 2 Department of Bioprocess and Polymer Engineering, Universiti Teknologi Malaysia, Johor Bahru 81310, Malaysia; 3 Department of Bioprocess Technology, Faculty of Biotechnology and Science Biomolecule, Universiti Putra Malaysia, Serdang 43400, Malaysia; 4 Department of Chemistry, Faculty of Resource Science and Technology, Universiti Malaysia Sarawak, Kota Samarahan 94300, Malaysia; 5 Department of Crop Science, Faculty of Agricultural Science and Forestry, Universiti Putra Malaysia, Bintulu Sarawak Campus, Bintulu 97008, Malaysia

**Keywords:** smart food packaging, pH-responsive, colour indicator, *Brassica oleracea*, sago starch, biopolymer film

## Abstract

To meet the need for food products to be safe and fresh, smart food packaging that can monitor and give information about the quality of packaged food has been developed. In this study, pH-sensitive films with sago starch and various anthocyanin concentrations of *Brassica oleracea* also known as red cabbage anthocyanin (RCA) at 8, 10, 12, and 14% (*w*/*v*) were manufactured using the solvent casting process. Investigation of the physicochemical, mechanical, thermal, and morphological characteristics of the films was performed and analysed. The response of these materials against pH changes was evaluated with buffers of different pH. When the films were exposed to a series of pH buffers (pH 3, 5, 9, 11, and 13), the RCA-associated films displayed a spectacular colour response. In addition, the ability of the starch matrix to overcome the leaching and release of anthocyanins was investigated. Higher concentrations of RCA can maintain the colour difference of films after being immersed in a series of buffer solutions ranging from acidic to basic conditions. Other than that, incorporating RCA extracts into the starch formulation increased the thickness whereas the water content, swelling degree, tensile strength, and elongation at break decreased as compared to films without RCA. The immobilisation of anthocyanin into the film was confirmed by the FTIR measurements. The surface patterns of films were heterogeneous and irregular due to the presence of RCA extract aggregates, which increased as the extract concentration enhanced. However, this would not affect the properties of films. An increase in thermal stability was noted for the anthocyanin-containing films at the final stage of degradation in TGA analysis. It is concluded that RCA and sago starch formulation has great potential to be explored for food packaging purposes.

## 1. Introduction

Food preservation is greatly aided by packaging, which protects food against conditions that cause chemical, physical, and microbiological deterioration while also ensuring food safety and quality [1]. Food packaging should also be appropriate to minimise negative environmental effects. The use of plastic packaging imposes a potential danger to human health which might arise from the uptake of food items that have come into touch with plastic or contain microplastic [2]. A deficit in petroleum resources also limits the growth of the plastics industry. Therefore, it is important to develop alternatives to plastics. Natural polymers of plant origin, such as polysaccharides, are the preferred choice for plastic replacement due to their abundance, non-toxicity, biodegradability, high stability, compatibility, and easy control of physicochemical properties through blending with other biopolymers. Starch can be used for food packaging purposes as starch is more compatible with some biopolymers, natural antioxidants and nanofillers, facilitating the development of a wide range of active edible films for various food packaging applications [3]. Numerous sources of starch, such as corn, potato, cassava, and wheat starch, have been investigated [4,5,6,7]. However, there are relatively few publications on sago starch produced from the sago palm, which is native to Southeast Asia. Sago starch has distinctive qualities all its own, including high viscosity and ease of moulding. It is also simple to gelatinise. Sago starch has developed into a valuable source for foodstuffs and industrial raw materials due to its low cost and wide availability [8].

Food packaging has also received a lot of attention in response to consumers’ growing concerns about accurate information on the quality of their food due to the uncertainty of food quality inside the packages. This situation has caused an increase in the amount of food waste that always occurs for conventional plastic packaging [9]. Therefore, the demand for smart packages is growing because they can recognise, sense, track, record, and display characteristics, providing consumers with accurate information about food conditions and storage environments [10]. Thus, smart packaging film can act as a spoilage indicator because it could contribute a significant role in overcoming food from being thrown away as waste throughout the supply chain [11]. Moreover, people need to be extra cautious with perishable foodstuff such as fresh milk since it starts showing signs of spoilage when the pH decreased to 5.98, signalling the formation of lactic acid from the initial pH value of 6.21. In addition to that, fresh meat starts to spoil when the pH increased to 6.47 from the initial pH of 5.72 due to the decomposition of several substances, which consequently produce ammonia and several amino sugars within the meat. In a brief, food spoilage can be indicated by modifications in pH as a response to the growth and metabolism of microorganisms or the development of organic acids and/or volatile amines [12].

It also communicates with the consumers and can help to inform them directly about the freshness of foodstuff, which is a very convenient and visual method. Hence, food package manufacturers must consider all these elements to optimise sustainability and mitigate consumers’ misconceptions about the safety of food products. Furthermore, the expiration date on the container is not the only way to determine the quality and freshness of food because it can also be impacted by incorrect handling and temperature [13]. Synthetic colourants are toxic and therefore natural dyes are used as replacements in biodegradable smart packaging and pH sensing. Anthocyanins are among the naturally occurring plant pigments that are particularly sensitive to different ranges of pH and easily change their colour in response to pH solutions due to their exceptional structure [8,9].

Anthocyanin belongs to the family of flavonoids from plants, fruits, and cereals [14]. The properties of anthocyanin which is hydrophilic and compatible with starch formulation make it a suitable candidate to be applied in the smart packaging materials [15]. There are several studies about the use of red cabbage (*Brassica oleracea)* as a pH indicator for smart packaging systems. However, there is a need to study to determine how the sago starch film matrix controls the release of red cabbage anthocyanin (RCA) and its ability to respond to different pHs. Anthocyanin extracted from *Brassica oleracea* can be considered an excellent candidate for natural indicators due to the high stability and chemical properties of the acylated anthocyanins [16]. This plant originated from Europe and is nowadays produced and harvested all over the world [17]. This study aims to develop starch-based films incorporated with *Brassica oleracea* anthocyanin and to determine the effect of anthocyanin concentration on the physical and mechanical properties. In addition, the response of smart films towards pH changes in a series of buffer solutions was determined.

## 2. Material and Methods

### 2.1. Materials

The commercial sago starch was bought from a local hypermarket in Bintulu, Sarawak. Red cabbage powder was obtained from Hana Natural company (Penang, Malaysia). Meanwhile, the glycerine used was supplied by HmbG Chemicals (Hamburg, Germany). The series of buffer solutions of pH 1.0–13.0 was bought from Bendosen Laboratory Chemicals (Bendosen, Norway).

### 2.2. Film Preparation

The red cabbage powder (8, 10, 12 and 14 g) was homogeneously stirred in 100 mL distilled water under magnetic stirring of a hotplate (Favorit, Model HS0707V2, Semenyih, Malaysia). Then, the solution was heated until mild boiling before added with 5 g of starch powder and 3 mL glycerol. The solution was then poured into a 20 cm × 20 cm casting plat and dried in an oven (Biuged, Guangzhou, China) at 60 °C for 3 h. After that, the dried film was peeled off the casting plate and sealed in a plastic container for further testing. The control film was prepared with sago starch, glycerol and distilled water without RCA being added to the film solution. Table 1 summarises the amount of anthocyanin being used throughout the preparation of the films.

### 2.3. Colour Change with pH Buffers

Colour changes of RCA solution or indicator solutions were observed by withdrawing 20 mL of RCA extract into tubes containing 20 mL of different pH solutions. A series of pH buffer solutions of pH were prepared to observe and quantify the discolouration of the indicator solutions. The standard or reference for indicator colour solutions is the solution of red cabbage extract (8% *w*/*v*) without the addition of buffer solution. The data collection was performed by using a Colour Reader CR10 (Konica Minolta CR10, Tokyo, Japan), and denoted as CIELAB colour scale: L* = 0 (black) to L* = 100 (white), –a* (greenness) to +a* (redness), and –b* (blueness) to + b* (yellowness). A positive sign (+) in L* values indicate a light colour, whereas it indicates a redder and yellower colour in a* and b* values, respectively. A negative sign (–) indicates greener and bluer colours in a* and b* values, respectively. Colour difference, often known as Delta (Δ), is a numerical comparison of a sample’s colour to the standard. The quantitative comparison of a sample’s colour to the standard is known as colour difference, which indicates the differences in absolute colour coordinates and is referred to as Delta (Δ). The total differences in colour (ΔE) were calculated to the equation:

(1) $$\Delta E=\sqrt{{\left(L_{0}^{*}-L_{sample}^{*}\right)}^{2}+{\left(a_{0}^{*}-a_{sample}^{*}\right)}^{2}+{\left(b_{0}^{*}-b_{sample}^{*}\right)}^{2}}$$

where ΔE is the colour difference; L*, a* and b* are the colour parameters of lightness, red–green chromaticity index, and yellow–blue-chromaticity indexes, respectively; *L*_0_, *a*_0_, and *b*_0_ are referring to the colour parameters of the RCA solutions (8% *w*/*v*) as the standard.

### 2.4. Characterisation of Films

#### 2.4.1. Colour Measurement of Films

In this study, the sago starch incorporated with RCA (SA-8%, SA-10%, SA-12%, and SA-14%) was used to measure the colour change at various pH levels. After drying in the oven, strips of film were cut into rectangles (3.0 cm × 3.0 cm) and soaked in the pH buffer solutions (pH 3, 5, 9, 11, and 13) for 60 min. The change of film’s colour intensity due to the release of anthocyanins into the buffer solutions was recorded using a Colour Reader CR10 (Konica Minolta CR10, Tokyo, Japan) and measured using the CIELAB colour space. The total colour difference (ΔE) was calculated based on the colour of the original films at each concentration. The colour changes of films after one hour were also recorded to determine the stability of films and the release of anthocyanin from the starch matrix. The colour difference between the original fresh film and the after immersion in a series of buffers was compared using Equation (1). The colour change of films indicates that the anthocyanins incorporated are responsive to the buffer solution and are released from the films and into the solution after a certain length of time.

#### 2.4.2. Moisture Content and Solubility and Swelling Degree

The film samples’ water content, water solubility, and swelling level were assessed according to the method by Sohany et al. [18] with a few minor modifications. Each of the films was cut (10 mm × 30 mm) and the initial weight, M_1_ was recorded before they were dried at 70 °C for 24 h in the oven. Then, the initial dry matter, M_2_ was obtained by taking the weight of the dried films. Each film sample was then soaked overnight at room temperature in distilled water, dried with filter paper, and weighed, M_3_. The samples were then dried again in an oven at 70 °C for 24 h to obtain the final dry weight of the undissolved material, M_4_. For each film sample, duplicate measurements were made to determine the average value of the parameters. These are the equations to determine the water content, water solubility, and swelling degree of films:

(2) $$\mathrm{Water}\;\mathrm{content}\;\left(\%\right)=\left(M_{1}-M_{2}\right)/M_{1}\times 100$$

(3) $$\mathrm{Solubility}\;\left(\%\right)=\left(M_{2}-M_{4}\right)/M_{2}\times 100$$

(4) $$\mathrm{Swelling}\;\mathrm{degree}\;\left(\%\right)=\left(M_{3}-M_{2}\right)/M_{2}\times 100$$

#### 2.4.3. Thickness and Mechanical Properties

Film thickness was measured by digital micrometre with 0.001 mm resolution (Insize, model IP54, Hanoi, Vietnam) with a precision of 0.001 mm at ten random positions around the strip film. The mechanical analysis of the films was performed using a CT3 Texture Analyser (AMETEK Brookfield, Massachusetts, USA) which complies with the standard ASTM D882. The samples were clamped between the tension grips and secured using double-sided adhesive tape. For the analysis, a constant rate crosshead speed of 1 mm/s was used. To verify the accuracy of the results, the results were computed using the average of five replications.

#### 2.4.4. Fourier Transform Infrared (FTIR)

FTIR spectra were obtained using a Fourier-transform infrared (FTIR) spectrophotometer (Perkin Elmer, Spektrum 100, Waltham, MA, USA). The analysis was performed with a resolution of 4 cm^−1^ in the infrared region between 4000 and 500 cm^−1^ from 32 scans.

#### 2.4.5. Scanning Electron Microscopy (SEM)

The images of surface films were recorded using an S-4800 scanning electron microscope (Hitachi Co., Ltd., Matsuda, Japan) at an accelerating voltage of 5 kV.

#### 2.4.6. Thermogravimetric Analysis (TGA)

The thermal degradation profile of the control film and anthocyanin-starch films was analysed using a simultaneous thermal analyser (STA) (Perkin Elmer, Waltham, MA, USA). Samples ranging from 5 g to 12 g with 125–250 μm particle size were placed in a crucible and heated at varying heating rates (20 °C/min, 30 °C/min, 40 °C/min) from 30 °C to 600 °C.

## 3. Results and Discussion

### 3.1. Colour Variation of RCA in Different pH Buffer Solutions

**Figure 1:**
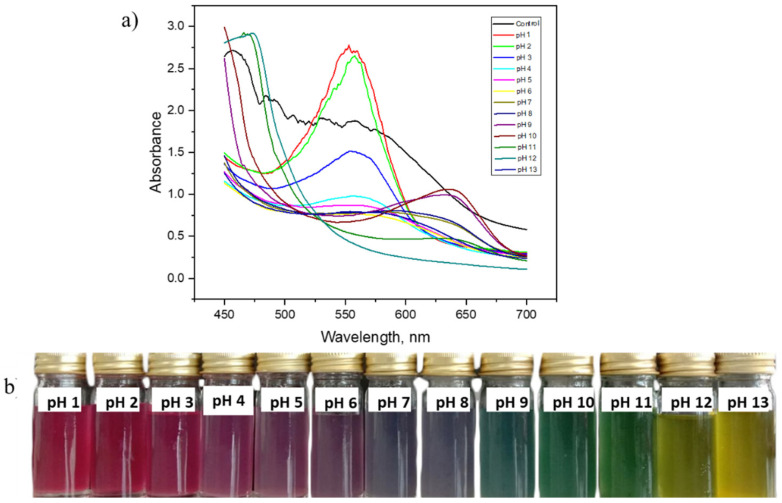
(**a**) UV–vis spectra of indicator solutions in a series of pH buffers and (**b**) changes of RCA solution in the buffer solutions.

The indicator solutions were introduced with a succession of buffer solutions (pH 1 to pH 13). The UV–vis spectra and absorbance peak of the red cabbage extract as control and indicator solutions in a series of pH buffers are shown in Figure 1a below. The RCA extracts in buffer solutions have shifted from acidic form (pH 1–5) to basic form (pH 8–13), which had the effect of shifting the maximum absorption peak from 550 to 630 nm. The shifting of spectra is most probably due to the conversion of chemical forms (flavylium cations to carbinol pseudobase) [19], which is in response to the change of the colours’ solution from red to yellow (Figure 1b).

After exposure to a variety of pH buffers, the RCA solutions changed colour dramatically as shown in Figure 1b. The solutions changed from purple to red colour when it was in contact with an acidic solution and became purplish when approaching a neutral pH. After the anthocyanin solution was mixed with basic buffer solutions, the solution became yellowish and greenish. The oxonium ion causes an extended conjugation of double bonds through three rings of aglycone moiety in an acidic solution, which aids photon absorption in the visible spectrum [20]. The primary flavylium cation has a structure that predominates between pH 1 and 3, which leads to purple and red colours under acidic conditions. The most prevalent form in the pH range of 4 to 5 is pseudobase carbinol, which is formed by the hydration of the molecule and results in a colourless indicator. The quinoidal basic purple colour structure was revealed when the pH was raised from 6 to 7 [15,16].

### 3.2. Characterisation of Films

#### 3.2.1. Colourimetric Analysis

Colour is one of the important physical properties of food packaging. The Colour parameters of RCA films were summarised in Table 2. The table shows the colour lightness value, L*, a*, and b* colour difference, and ΔE of different concentrations of anthocyanin incorporated in the sago starch solution. The control film which is the starch film without RCA has been used as the standard reference sample. The control film showed the highest lightness value, L*, and lowest value for both a* and b* colour parameters. The RCA films with the addition of RCA showed that the lightness had decreased due to the high concentration of anthocyanin whereas the a* and b* parameters are both decreased. The colour difference of the RCA films from the control film has also increased. As can be seen from the table, SA-14% has the highest colour difference from the standard film due to the high amount of red cabbage extract. The lightness, L* of the SA-14% is also lower due to the dark appearance of the film.

#### 3.2.2. The Physical Appearance of Films

**Figure 2:**
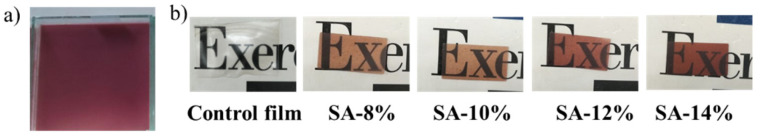
(**a**) The mixture solution cast into plate; (**b**) control film and RCA film physical appearance at four different concentrations.

The control film and RCA-containing films were cast onto an acrylic plate before being oven-dried for 3 h (Figure 2a). As shown in Figure 2b, the colours of starch incorporated with RCA films were significantly different from that of control film due to the addition of anthocyanin. The control film showed a translucent white whereas the RCA films presented translucent purplish colour. As the concentration of RCA increased, the translucency of the films became decreased. The low translucency of the RCA-containing films will not affect the decision making on the food quality by the consumers and food sellers since only a small strip of the film will be used on top of the food packaging to indicate the spoilage of the food. Many of these consumers want information that gives them confidence in the packaging and labels that are essential to their purchase [19]. High loading of RCA extract in the starch films also caused the thickness to increase which also consequently decreases the opacity of the RCA films. The purple colour of RCA films could be attributed to anthocyanins extracts in the starch solution as shown in Figure 2a.

#### 3.2.3. Thickness

In addition to that, there is an increase in film thickness with increasing red cabbage concentration. When the drying process of the starch solution occurs, the thickness has reduced about two-fold since the moisture has evaporated and the film becomes compact. Thus, the amount of red cabbage extract affects the thickness of the films. From Table 3, the thickness values ranged from 0.12 mm to 0.23 mm. The control film (without red cabbage extract) has the lowest thickness and the films with incorporated with RCE have increasing thickness. Xue Mei et al. [21] stated that the thickness of the films created will be increased when a larger concentration of torch ginger extract (TGE) was added to the film.

#### 3.2.4. Water Content, Solubility, and Swelling Degree

Table 3 displays that the water content (WC) value of starch incorporated with RCA films is lower than that of control. The interaction between the hydroxyl group of anthocyanins and starch in RCA extract lowered the amount of free hydroxyl group interactions with moisture. The anthocyanins reduced the pores in the starch matrix, which in turn decreased the moisture content of the biofilms. Xue Mei et al. [21] claimed that after a higher concentration of torch ginger extract (TGE) was added, the moisture content of films increased, as well as the thickness of films. This might be because of the hydroxyl groups being available from the interaction of the sago starch, glycerol, and TGE. However, this study finds that films’ thickness increased whereas the water content decreased when incorporated with RCA extracts. There is also a study stating that the addition of anthocyanin does not affect the water content of films [22].

The water solubility (WS) and swelling degree (SD) of the films are different with different loading percentages of red cabbage extracts in the films, as shown in Table 3. The films were swollen after they were soaked in water overnight due to the water absorption ability of the films. It is shown that SA-14% film has the highest water solubility compared with other concentration percentages. The high water solubility properties of biofilms can be adopted as edible coating films, which can be applied to food surfaces [23]. However, the swelling degree has decreased significantly with the addition of RCA extract until sample SA-12% film. The swelling degree raised again for sample SA-14%, Higher swelling in the films may be the result of an increase in the hydrophilic group [24]. The swelling of granules, which is described as an initial step of starch processing, is largely influenced by the granule shape, amylose–amylopectin enlargement, and their interactions [25]. According to Halász & Csóka [9], the higher swelling degree is favourable as an aqueous medium in which changing pH can infiltrate the bulk matrix and enhance the pH sensitivity.

From Table 3, a higher concentration of RCA incorporated in films results in an increase in water solubility and a decrease in swelling degree. Since pH indicator films have a high-water solubility, one method for utilizing them as feasible intelligent packaging is to minimise the contact time with food that contains a lot of water, such as meat. The films can then be mounted to the top of the package or in the headspace of the packaged food/drinks and only detect the volatile chemical compounds inside the packaging.

#### 3.2.5. Mechanical Properties

**Figure 3:**
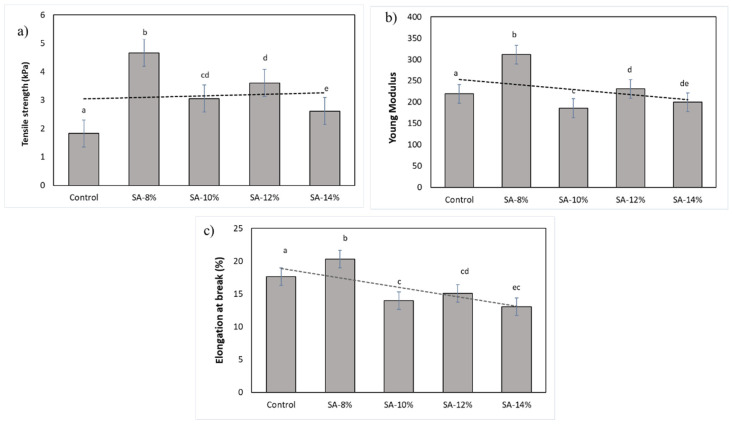
Mechanical properties of the RCA incorporated and control films: (**a**) tensile strength; (**b**) Young Modulus, and (**c**) elongation at break.

Tensile strength is the ability of plastic material to sustain the highest amount of tensile stress while being pulled or stretched without rupture. Figure 3 displays the mechanical properties of the starch film (0%) and anthocyanin incorporated in starch films. Tensile strength (TS) and elongation at break (EB) value varied in the range of 1.8–4.8 kPa and 13–20%, respectively. It is shown that the films had strengthened when anthocyanin was added to the starch matrices. This is proven when the tensile strength has increased by 60.8% from the incorporation of 8% of RCA extract into the film. This is supported by the previous result in Section 3.2.3 where the control film has a significant lowest thickness compared to the SA-8% film which probably caused the increase in the tensile strength.

However, anthocyanin has no significant effect when the concentration increased to 10% and above (Figure 3a). According to Yong et al. [26], the tensile strength steadily rose as the amount of purple eggplant extract (PEE) was increased with the addition of anthocyanin to chitosan (CS) films. The formation of hydrogen bonds between the hydroxyl/amino groups of CS and the polyphenols in the extracts could account for the improved tensile strength of films containing PEE [26]. The elongation at break or also known as fracture strain of anthocyanin incorporated in starch films has reduced significantly as compared to the control film. In this study, the elongation at break increased as the concentration increased. The Young’s modulus also decreased as the RCA concentration increased. These results indicate that the RCA films have become more elastic due to higher EB values. It is also reported smart film added with anthocyanin showed improved elasticity and lower tensile strength [27].

In addition, this result agrees with S. Roy et al. [28] who reported that the inclusion of anthocyanins in biopolymer-based films improved tensile properties while reducing elongation at break. In addition, the film synthesised from the mixed hydrogels made of poly (vinyl alcohol) (PVA) solution, chitosan (CS) solution, and sodium tripolyphosphate (STPP), was discovered to have excellent mechanical properties and exhibit an accelerated change in colour in pH buffers, as reported by Vo, T et al. [29]. As compared with the starch film, bayberry extract (BBE) incorporated in starch films exhibited higher thicknesses and tensile strength [30]. Zhang et al. [31] also reported that red cabbage extract enhanced the mechanical characteristics because of the potential for intermolecular interactions between the anthocyanins in the extracts and the film-forming matrix.

#### 3.2.6. Chemical Characterisation by Using Fourier Transform Infrared (FTIR)

**Figure 4:**
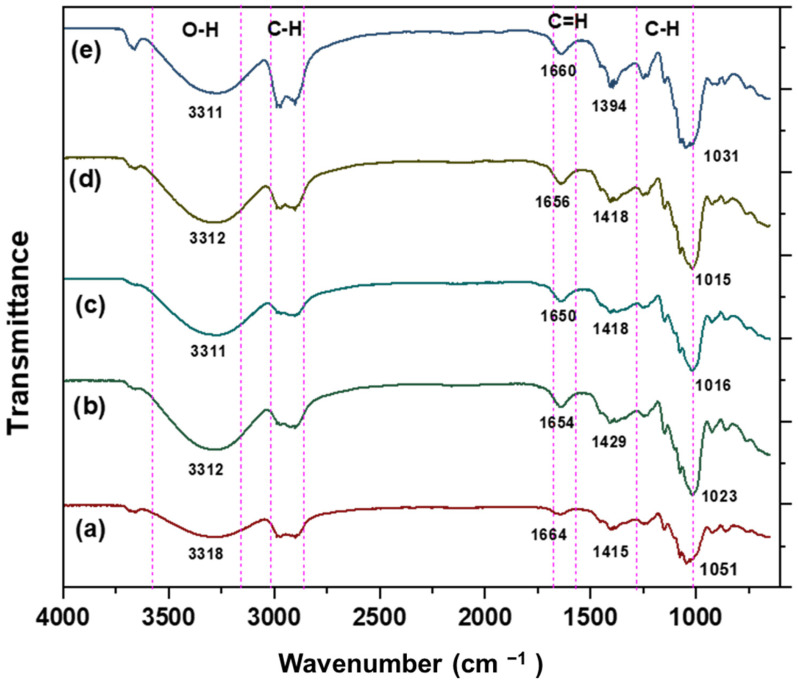
FTIR spectra of starch films and anthocyanin incorporated in starch films, (**a**) control film, (**b**) 8% RCA film, (**c**) 10% RCA film, (**d**) 12% RCA film and (**e**) 14% RCA film.

In this study, FTIR was used to examine the interactions that could occur between the film components, as well as the type of interactions. Figure 4 demonstrates the FTIR spectra of the different starch-based films. It shows that anthocyanin incorporated in starch films (8, 10, 12, 14 and 16%) has a similar spectrum to the control (starch film). Each film demonstrated a broad band around 3100 to 3600 cm^−1^ which was ascribed to the strong stretching vibration of a substantial hydroxyl group (O–H) present in starch, glycerol, water [18], and anthocyanin [23,27].

The FTIR spectrum for RCA films shows a strong absorption band with a maximum at 1023 cm^−1^ (8%), 1016 cm^−1^ (10%), 1015 cm^−1^ (12%), and 1031 cm^−1^ (14%) corresponding to aromatic ring C-H deformation. Concurrently, withinside the spectra of anthocyanin biofilms, a strong absorption between peaks at 1600 cm^−1^ and 1700 cm^−1^ corresponds to the bending vibration of C=H aromatic rings. On the other hand, the peak within the range 1633 to 1647 cm^−1^ represents the C=C aromatic ring indicating the presence of aromatic compounds in the extract, bands from 1407.3 to 1149.3 cm^−1^ arise from the oscillations of the C–O–C group [32]. Eskandarabadi et al. [33] stated that the characteristic peaks related to the aromatic and phenolic structure of the anthocyanin can be seen at 3250 cm^−1^, 1400–1600 cm^−1^, and 1300–1400 cm^−1^.

#### 3.2.7. Micrograph Study by Using Scanning Electron Microscopy (SEM)

**Figure 5:**
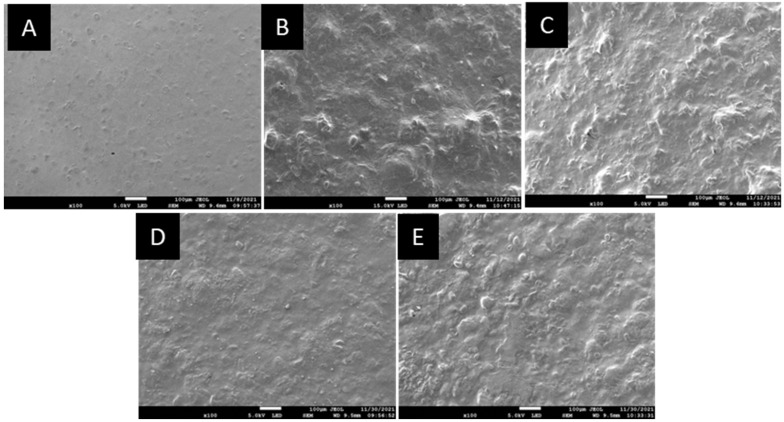
SEM micrograph of (**A**) 0%; (**B**) 8%; (**C**) 10%; (**D**) 12%; and (**E**) 14% *w*/*v* of red cabbage extract incorporation.

SEM images of the samples were taken to examine and visualise the microstructural changes of the starch film and the starch film incorporating the RCA extract (Figure 5). It could be observed that the control film (Figure 5A) was compact, smooth, and had a continuous surface. However, starch films incorporated with RCA at a concentration of 8, 10, 12, and 14% showed that the surface is uneven with the addition of RCA. Surface segregation of RCA-containing films showed that the extract is not completely solubilised in the starch solution. It can be seen that the size of segregation becomes smaller as the RCA concentration increases in starch films.

Coarser microstructures were observed in the films that incorporated 8, 10, 12 and 14% *w*/*v* of RCA (Figure 5B–E) can be attributed to the anthocyanin molecules deposition on the surface of the material, as reported by Carvalho et al. [34]. Luchese et al. [35] reported that surface roughness did not affect film colour change as a function of pH indicator. According to Prietto et al. [36], insufficient interaction of anthocyanin compounds with starch and glycerol can lead to rough films.

#### 3.2.8. Thermal Characterisation by Using Thermogravimetric Analysis (TGA)

**Figure 6:**
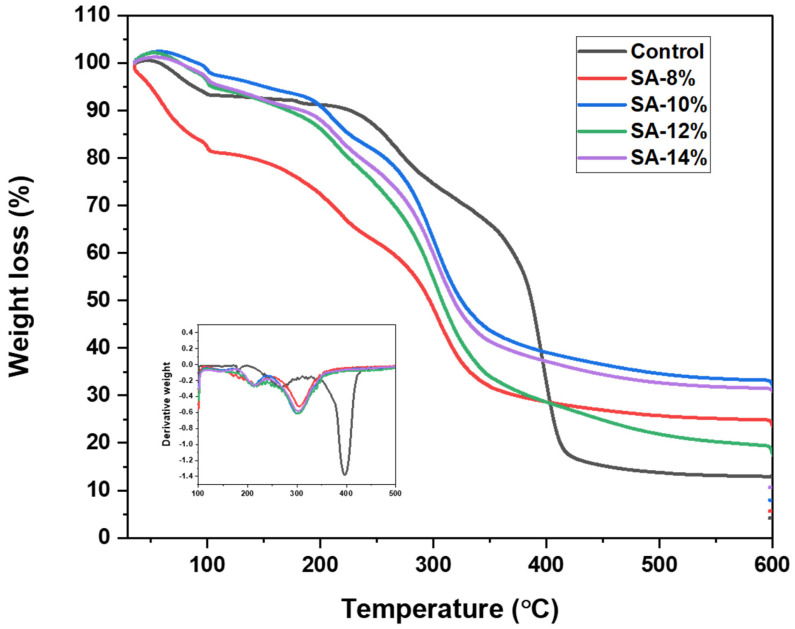
TGA curve of a control film and anthocyanin incorporated in starch films; SA−8%, SA−10%, SA−12%, and SA−14%.

Thermogravimetric analysis of starch-based films with different concentrations of RCE was performed to investigate their thermal stabilities and degradation profiles. Figure 6 exhibits the thermal degradation of starch films and films incorporated with anthocyanins. TGA revealed an almost similar degradation/decomposition profile following three main steps for all the films. There is also a study by Amin et al. [37] who stated that the TGA curve of starch thermoplastic starch films appeared to have a two thermal degradation pattern. From the figure, the first stage exposed around 7 to 20% weight loss between 35 to 100 °C which was attributed to water loss from all the film samples. The first peak temperature corresponds to the evaporation of the bound water and low molecular weight compounds in the films [22]. Luchese et al. [35] also mentioned that for cassava starch containing blueberry pomace, a slight weight loss near 100 °C may be due to evaporation of the water content of the sample, whereby Qin et al. [24] reported that the first degradation step occurs at the temperature range 30 to 185 °C for films based on cassava starch and anthocyanins from *Lycium ruthenicum* Murr.

The second decomposition stage was exhibited at around 100 to 300 °C as shown in Figure 6, attributed to the glycerol-rich phase volatilisation and degradation. Lozano-Navarro et al. [38] reported that the second degradation at 290 °C of chitosan-starch films with anthocyanin was due to the decomposition temperature of glycerol. Zhang et al. [32] also mentioned that a sharp mass loss between 280 and 350 °C was caused by the starch chain decomposition. Further heating above 300 °C will induce the main thermal degradation, which reflected the major mass loss due to hydroxyl dehydration. The start of the third decomposition stage is slightly varied among all the films. Figure 6 also shows that the control film exhibited higher thermal stability than that of the RCA-containing films. Then, the major weight loss reduction occurred at the third stage due to the hydroxyl dehydration [18]. Jumaidin et al. [39] who worked on sugar palm starch, have reported that this stage was ascribed to the elimination of hydrogen groups, decomposition, and depolymerisation of the starch carbon chains. At 600 °C, the final weights of the control film, SA-8%, SA-10%, SA-12% and SA-14% films were 13.0, 24.9, and 33.3, 19.6 and 31.6% of the original weight, respectively.

The DTG curve peaks depict the sample’s maximum rate weight of loss as shown in Figure 6. The curve revealed that anthocyanin affected the maximum weight loss rate of the films, and the rate was higher for the control films. Table 4 shows the derivative thermogravimetric (DTG) peak temperature and percent weight loss of the films at the third stage. However, in this study, the film’s stability at temperatures below 100 °C supports the suitability of the films for use in food packaging applications.

### 3.3. Colour Responses of pH-Sensitive Films

The colour parameters or CIELAB coordinates (L*, a*, and b*) of the films in various pH solutions were summarised in Table 5. It was observed that the film changes after immersion in a wide range of pH buffers. The original colour of the RCA films changed after an hour as the colour of the films became pale and lighter since the anthocyanin is released into the buffer solution. The higher concentration of anthocyanin in starch films showed a lower release into the solution since the changed colour pigments which indicates the anthocyanin was still dark despite its change based on the pH of buffers. This result also indicates that higher RCA concentration can retain the release after one hour. The swelling degree affects the colour change of films in liquid buffer solution. From Table 2, the highest concentration of RCA (SA-14%) contributes to the highest swelling degree.

The trend of film colour change was similar to that of the RCA extract’s solution, which gradually changed from pink (pH 3–5) to green (pH 9–11) and yellow (pH 13). Additionally, SA-14% film exhibited a bolder colour than SA-8% due to its abundant anthocyanin content. Colour parameters (L*, a*, and b*) were used to distinguish colour differences. Table 5 also shows that the lightness and yellowness to blueness, b* of 8% of RCA films are higher than 14% of RCA films. The positive a* value marked the redness of the sample, while the negative a value marked the greenness of the sample. Structural changes of anthocyanins at different pH values caused film colour changes. It was attributed to the conversion of flavylium cations to anionic quinoids in acidic conditions as the pH shifts to alkaline. These results indicated that the pH-sensitive films in this study possessed a pronounced colour response towards different pH values.

**Figure 7:**
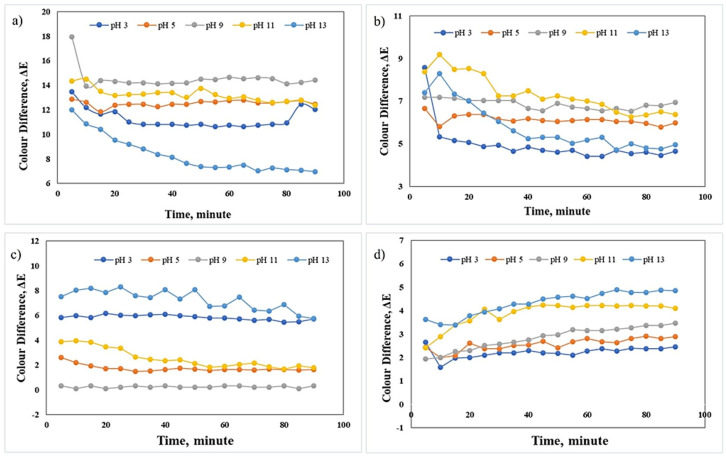
The colour changes of films (**a**) SA−8%, (**b**) SA−10%, (**c**) SA−12%, and (**d**) SA−14% at different pH of buffer solutions.

It was reported that leaching of colourant or anthocyanin release that could take place over time led to inaccurate responses or false positive indications [40]. Figure 7 shows the trend of colour difference changes every five minutes for each RCA containing films. It was observed that the colour difference of SA-12% and SA-14% displayed a small fluctuation at the beginning of sampling time before the values become equilibrium until the end of sampling (90 min). However, the trend for SA-8% and SA-10% showed a significant decrease in colour difference before the values become equilibrium. The colour of anthocyanins is depending on the pH of the solution [41]. This result indicates that the higher amount of anthocyanin loading in the starch films slows down the release of anthocyanin from film and makes the film maintains its colour intensity. Thus, the decreasing intensity of the colours also means that the anthocyanin has been mobilised to the buffer solution which happens to the sample films SA-8% and SA-10%. This result also related to the solubility and swelling degree for each RCA containing film as a higher percentage of solubility and swelling degree of SA-14% making the film able to maintain the small colour difference after a while.

## 4. Conclusions

Food spoilage is a process or change that makes a product unappealing or unfit for ingestion. Hence, smart packaging that can alert consumers about harmful foods is beneficial for human beings. Natural indicators such as red cabbage (*Brassica oleracea*) anthocyanins exhibit a significant colour change between acidic and alkaline conditions (about pH 6 and 9) and are suitable to be applied as smart food packaging. In addition, the ability of the high concentration of RCA-containing films to retain the colour intensity after sensing the change of pH for a longer time provides an effective way to express the quality of food. The natural dye pigments from this plant are particularly well-suited to detecting a wider range of pH variations that deteriorate due to pH fluctuations. They have a wider range of discolouration, ranging from red to purple between pH 3 and 5 for acidic food products and pH 7 to 9 for basic food products. Therefore, the incorporation of anthocyanin from *Brassica oleracea* into starch film was an appropriate technique for making colour indicator film packaging since anthocyanin is hydrophilic and soluble in water. The mechanical properties of the indicator films were also improved after the addition of anthocyanin. Moreover, SEM micrographs result indicated that the RCA extract did not alter the spatial structure between the starch structures. Thereby, colourimetric pH sensor films could be commercialised by incorporating sago starch and anthocyanin extracted from *Brassica oleracea* to serve as sensors that respond rapidly and inform customers about the condition and quality of the packaged food. Even while anthocyanin-based indicators can accurately predict the food shelf-life in a variety of products at a lab scale, the manufacturing processes for this kind of sensor still limit their industrial output [42]. In addition, it is suggested that some modifications need to be completed on this starch-based film to control the release of anthocyanin by the addition of other natural resources to hold the anthocyanin longer in the starch matrix [43]. In addition, studies related to food packaging such as using food simulants could be added for future studies.

## Figures and Tables

**Table 1:** Amount of anthocyanin being used throughout the preparation of each film.

Films	g/L
Control	0
SA-8%	80
SA-10%	100
SA-12%	120
SA-14%	140

**Table 2:** Colour parameters including *L, a, b,* and ΔE of control and RCA-containing films.

Films	L*	a*	b*	Δ*E*
Control	45.27	0.57	0.6	0.0
SA-8%	42.58	3.83	2.86	4.80
SA-10%	39.56	3.59	1.82	6.57
SA-12%	39.31	3.52	1.13	6.67
SA-14%	38.52	2.69	0.75	7.08

**Table 3:** Thickness, water content, water solubility, and swelling of films.

Sample	Thickness (mm)	Water Content (%)	Water Solubility (%)	Swelling Degree (%)
Control	0.12 ± 0.04 ^a^	40.38 ± 8.39 ^a^	32.37 ± 6.96 ^a^	130.31 ± 23.69 ^a^
SA-8%	0.17 ± 0.01 ^b^	31.39 ± 1.68 ^ab^	56.62 ± 11.73 ^b^	74.80 ± 53.68 ^b^
SA-10%	0.19 ± 0.01 ^bc^	30.17 ± 0.79 ^bc^	55.60 ± 5.26 ^bc^	79.46 ± 46.06 ^bc^
SA-12%	0.22 ± 0.02 ^d^	29.40 ± 0.42 ^cd^	57.95 ± 8.81 ^cd^	79.52 ± 53.35 ^cd^
SA-14%	0.23 ± 0.02 ^de^	23.95 ± 0.32 ^e^	59.45 ± 0.01 ^de^	99.48 ± 3.09 ^e^

Reported values for each film are means ± standard deviation (*n* = 5 for thickness, *n* = 3 for WC, WS, and SD). Different letters in the same column (^a^, ^b^, ^c^, ^d^, and ^e^) indicate significant differences between samples (*p* < 0.05), according to Tukey’s test.

**Table 4:** Derivative thermogravimetric (DTG) peak temperatures and percent weight loss.

Samples	Peak Temperature (°C)	Weight Loss (%)
Control film	190.83	91.38
SA-8%	301.89	47.62
SA-10%	299.91	63.31
SA-12%	301.85	53.32
SA-14%	302.61	58.34

**Table 5:** The colour spectrum of the pH-sensitive films containing red cabbage as a function of pH.

Sample	pH	Colour	L*	a*	b*	ΔE
SA-8%	3	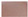	46.9	4.3	0.8	10.7
	5	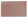	45.1	1.6	1.1	12.8
	9	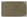	43.6	0	0.9	14.6
	11	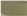	45.2	0.2	2.0	12.9
	13	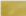	51.9	0.1	5.2	7.31
SA-10%	3	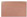	45.1	4.9	0.7	4.4
	5	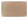	43.6	2.2	0.7	6.1
	9	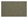	44.2	0.1	0.8	6.7
	11	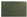	43.7	−0.2	1.8	7.0
	13	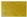	51.2	0.1	4.8	5.0
SA-12%	3	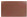	42.6	5.6	0.1	5.8
	5	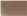	44.6	2.0	0.8	1.6
	9	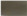	44.8	−0.2	0.5	0.3
	11	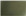	44.2	−0.2	2.0	1.9
	13	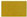	47.3	0.5	7.3	6.8
SA-14%	3	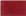	43.0	3.9	0.6	2.3
	5	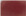	43.2	2.1	0.8	2.8
	9	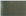	42.0	0.6	0.5	3.2
	11	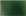	40.7	−0.7	0.3	4.1
	13	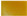	42.6	1.8	4.4	4.6

## Data Availability

Not applicable.
